# Leadership and job satisfaction in the Mobile Emergency Care Service context*

**DOI:** 10.1590/1518-8345.3455.3260

**Published:** 2020-05-11

**Authors:** André Almeida de Moura, Andrea Bernardes, Alexandre Pazetto Balsanelli, Carina Aparecida Marosti Dessotte, Carmen Silvia Gabriel, Ariane Cristina Barboza Zanetti

**Affiliations:** 1 Secretaria Municipal de Saúde, Serviço de Atendimento Móvel de Urgência, Rio Verde, GO, Brazil.; 2 Universidade de São Paulo, Escola de Enfermagem de Ribeirão Preto, PAHO/WHO Collaborating Centre for Nursing Research Development, Ribeirão Preto, SP, Brazil.; 3 Universidade Federal de São Paulo, Escola Paulista de Enfermagem, São Paulo, SP, Brazil.

**Keywords:** Leadership, Nursing, Job Satisfaction, Emergencies, Personnel Management, Emergency Medical Services, Liderança, Enfermagem, Satisfação no Trabalho, Emergências, Administração de Recursos Humanos, Serviços Médicos de Emergência, Liderazgo, Enfermería, Satisfacción en el Trabajo, Urgencias Médicas, Administración de Personal, Servicios Médicos de Urgencia

## Abstract

**Objective:**

to evaluate the correlation between the practice of Coaching Leadership performed by nursing coordinators and job satisfaction, in the self-perception of coordinators and the perception of nursing technicians of the Mobile Emergency Care Service.

**Method:**

a descriptive, correlational study that used the Questionnaire on Self-Perception of the Nurse in the Exercise of Leadership to measure the self-perception of leadership of the eleven nursing coordinators and the Questionnaire on Nursing Technician Perception in the Exercise of Leadership to verify the perception of 155 nurse technicians. The Job Satisfaction Questionnaire assessed job satisfaction in both categories. Correlations among instrument domains were determined using the Spearman test (p<0.05) and the association was analyzed.

**Results:**

the Coaching Leadership exercise correlated with job satisfaction (p-value=0.001), both in the self-perception of nursing coordinators, with high correlation (Spearman coefficient - (0.835), and in the perception of nursing technicians, with moderate association (Spearman coefficient - 0.678).

**Conclusion:**

coaching Leadership showed a positive correlation with job satisfaction, marked by mutual trust, continuous interaction between nurse and nursing technicians, and the pursuit of professional and personal development.

## Abstract (pt)

**Objetivo:**

avaliar a correlação entre a prática de Liderança *Coaching* exercida pelos coordenadores de enfermagem e a satisfação no trabalho, na autopercepção dos coordenadores e na percepção dos técnicos de enfermagem do Serviço de Atendimento Móvel de Urgências.

**Método:**

estudo descritivo, correlacional, que utilizou o Questionário de Autopercepção do Enfermeiro no Exercício da Liderança para mensurar a autopercepção da liderança dos onze coordenadores de enfermagem e o Questionário de Percepção do Técnico/Auxiliar de Enfermagem no Exercício da Liderança para verificar a percepção dos 155 técnicos. Já o *Job Satisfaction Survey* avaliou a satisfação no trabalho de ambas as categorias. Foram determinadas as correlações entre os domínios dos instrumentos por intermédio do teste de Spearman (p<0,05), sendo analisado o seu poder de associação.

**Resultados:**

o exercício da Liderança *Coaching* correlacionou-se com a satisfação no trabalho (*p*-valor=0,001), tanto na autopercepção dos coordenadores de enfermagem, com alta correlação (coeficiente de Spearman - 0,835), quanto na percepção dos técnicos de enfermagem, com moderada associação, (coeficiente de Spearman - 0,678).

**Conclusão:**

a Liderança *Coaching* apresentou correlação positiva com a satisfação no trabalho, marcada pela confiança mútua, interação contínua entre líder (enfermeiro) e liderados (técnicos de enfermagem) e a busca do desenvolvimento profissional e pessoal.

## Abstract (es)

**Objetivo:**

evaluar la correlación entre la práctica del *Coaching* de Liderazgo ejercida por los coordinadores de enfermería y la satisfacción laboral, en la autopercepción de los coordinadores y en la percepción de los técnicos de enfermería del Servicio Móvil de Atención de Emergencia.

**Método:**

estudio descriptivo y correlacional que utilizó el Cuestionario de Autopercepción del Enfermero en el Ejercicio de Liderazgo para medir la autopercepción de liderazgo de los once coordinadores de enfermería y el Cuestionario de Percepción de Técnico/Asistente de Enfermería en el Ejercicio de Liderazgo para verificar la percepción de 155 técnicos. Por otra parte, el *Job Satisfaction Survey* evaluó la satisfacción laboral en ambas categorías. Las correlaciones entre los dominios de los instrumentos se determinaron mediante la prueba de Spearman (p<0,05), y se analizó su poder de asociación.

**Resultados:**

El ejercicio de *Coaching* de Liderazgo se correlacionó con la satisfacción laboral (*p*-valor=0,001), tanto en la autopercepción de los coordinadores de enfermería, con una alta correlación (coeficiente de Spearman - 0,835), como en la percepción de los técnicos de enfermería, con asociación moderada (coeficiente de Spearman - 0,678).

**Conclusión:**

el *Coaching* de Liderazgo se correlacionó positivamente con la satisfacción en el trabajo, marcada por la confianza mutua, la interacción continua entre el líder (enfermero) y dirigidos (personal de enfermería) y la búsqueda de desarrollo profesional y personal.

## Introduction

Leadership is an indispensable competence for nurses’ professional practice in an increasingly globalized society and in a contemporary competitive labor market. It is from leadership and based on its precepts that the nurse guides the work process and administers the team to reach common goals^(^ 1 ^)^.

In addition, the quality of nursing care is influenced by effective nurse leadership, as this professional inspires and encourages the team to accept innovation and transformation actions. This practice encourages commitment and change, as well, as strengthens the trust bond between leader and team members, contributing to the quality of care provided, mediated by job satisfaction^(^ 2 ^-^ 3 ^)^.

Thus, it is noted that leadership creates a positive or negative work environment, depending on the model adopted to analyze this competence. Likewise, healthcare organizations lack leading nurses who anticipate problems and try to solve them through interaction with employees, positively impacting their satisfaction^(^ 4 ^)^.

In this perspective, it is worth mentioning two researches developed on the topic. In the first one, in a systematic review, 52 manuscripts were highlighted, with strong correlation between leadership styles and job satisfaction^(^ 5 ^)^. Conducted in Brazil, the second research pointed out that the new models of nursing leadership exert a positive relationship on job satisfaction, either indirectly - mediated by variables such as empowerment, supervised support and the supportive environment or directly - reaffirming the relevance of investment in the improvement and development of this competence in nursing professionals by health services^(^ 6 ^)^.

In this scenario, health organizations should invest in these new leadership models in order to enable assertive decision making, based on intense and dialogic communication, and thus transpose the evidence into practice and practice into evidence^(^ 7 ^)^. Therefore, it is important that theoretical references focused on contemporary leadership models not only based on researches, but also the exercise of this competence by the nurse, aiming at the instrumentalization of this professional and the promotion of positive influences in their work environment^(^ 8 ^)^.

Among the new models and contemporary leadership theories that have been adopted by nursing teams and health organizations, it is important to praise Coaching Leadership. Conceptually, it is the competency through which leaders seek to support the team in achieving results, to concurrently enable them to boost talent, develop other attributes and stimulate the potential of team members. In nursing, its exercise qualifies the role played by nurses in organizations, aiming to improve the quality of care provided to patients. The model is based on four aspects: communication, giving and receiving feedback, empowering and influencing and supporting the team to achieve organizational results^(^ 8 ^-^ 11 ^)^.

When analyzing the scientific production about Coaching Leadership, it is verified that there are still few studies in nursing that seek to analyze this competence, especially relating it to job satisfaction. Moreover, there are few studies that contemplate the various scenarios of nursing professionals, including the Mobile Emergency Care Service (SAMU- abbreviation in Portuguese)^(^ 6 ^)^.

SAMU is a key element of the National Emergency Care Policy, which describes the roles of nursing care professionals, as well as the coordinators of this class of professionals^(^ 12 ^)^. SAMU unit coordinators need to perform management actions on a daily basis to ensure the quality of care provided in emergency context. In this context, these nurses constantly seek the development of strategies to overcome the challenges^(^ 13 ^)^ that presuppose the exercise of some skills - such as leadership^(^ 14 ^)^- which, consequently, enable a favorable scenario for job satisfaction.

Based on the information above and considering the new models of leadership in nursing, this study aimed to evaluate the correlation between the practice of Coaching Leadership performed by nursing coordinators and job satisfaction, in the self-perception of coordinators and the perception of nursing technicians of the Mobile Emergency Care Service, in order to answer the following question: Is there a correlation between the practice of Coaching Leadership performed by nursing coordinators and job satisfaction, both in the self-perception of coordinators and in the perception of SAMU nursing technicians?

## Method

This is a descriptive correlational study conducted in eleven out of twelve SAMU macro regional headquarters units from the State of Goias, in which the regulation centers are located. One of the research members was responsible for applying the questionnaires to subjects who consented to participate, at dates and times previously scheduled between April to August 2017.

In this scenario, we sought to interview all nursing technicians and coordinators of these eleven units, constituting a population for convenience and not probabilistic. From the 221 workers in these categories under analysis, working in the units, 210 were nursing technicians and eleven coordinators.

Therefore, nursing coordinators and technicians in office for, at least, six months were included. In contrast, professionals from both categories who were on labor prerogatives (vacation, bonus leave or for health reasons) during data collection were excluded from the sample.

We used the instrument of characterization of subjects to obtain sociodemographic data. The data regarding Coaching Leadership were obtained from the scalar measurement questions related to skills and attitudes exercised by leaders and team members in the practice of this leadership model in the following instruments: Questionnaire on Self-Perception of the Nurse in the Exercise of Leadership (QUAPEEL - abbreviation in Portuguese) to the nursing coordinators and the Questionnaire on Nursing Technician Perception in the Exercise of Leadership (QUEPTAEEL - abbreviation in Portuguese) to analyze the perception of Coaching Leadership by the nurse technicians. Each questionnaire had 20 items, subdivided into four domains corresponding to Coaching Leadership: communication, giving and receiving feedback, empowering and influencing and supporting the team to achieve organizational results. Composed of a Likert scale, this is the presentation for each item: Never (1), Rarely (2), Not always (3), Almost always (4) and Always (5). The overall instrument score ranges from 0 to 100, with values closer to 0 corresponding to the lowest perception of Coaching Leadership practice and 100 to the highest perception. It is worth mentioning that the instruments were constructed and validated nationally, covering the context and nursing professionals, whose Cronbach’s alpha values for each instrument were 0.911 (QUAPEEL) and 0.932 (QUEPTAEEL)^(^ 9 ^)^.

Regarding job satisfaction, data were obtained using the Job Satisfaction Survey (JSS) instrument^(^ 15 ^)^, whose adaptation and cross-cultural validation for Brazil focused on minimizing gaps in the evaluation of job satisfaction in health researches, especially nursing, with Cronbach’s alpha equal to 0.92^(^ 16 ^)^.

This instrument has 36 fragmented items in the nine domains defined from a literature review on the dimensions of job satisfaction^(^ 15 ^)^: salary (items 1, 10, 19 and 28), promotion (items 2, 11, 20 and 33), supervision (items 3, 12, 21 and 30), benefits (items 4, 13, 22 and 29), rewards (items 5, 14, 23 and 32), operating procedures (items 6, 15, 24 and 31), employees (items 7, 16, 25 and 34), job nature (items 8, 17, 27 and 35) and communication (items 9, 18, 26, 36). In the JSS domains, there are four items on a six-point Likert-type scale, which ranks “Strongly Disagree”, “Moderately Disagree”, “Slight Disagree”, “Slight Agree”, ‘’Moderately Agree’’, and “Strongly agree”. This continuous variable of individual satisfaction can range from low (dissatisfied) to high (satisfied). Considering that there is no specific cutoff score that determines whether an individual is satisfied or dissatisfied, the scores for each domain ranged from 4 to 24 points, with the lowest scores attributed to less satisfied individuals. The values of the total score of the instrument ranged from 36 to 216 points, since the closer to the value of 216, the more the individual was satisfied about the work^(^ 16 ^)^.

Data were double entered in spreadsheets and after this step, the descriptive statistical analysis of the variables was performed using the statistical software SAS® 9.3 (Statistical Analysis System).

The exploratory analysis of sociodemographic data was made through measures of central position and dispersion, allowing the qualitative variables to be presented through absolute and relative frequencies and the quantitative described by means, medians, standard deviations, minimum and maximums values.

In addition, we used the Spearman test (nonparametric statistical test), so that the correlations among the variables in the study were interpreted, either positively or negatively, according to the power of the correlation: very strong (0.9 to 1.0 or -0.9 to -1.0), strong (0.7 to 0.9 or -0.7 to -0.9), moderate (0.5 to 0.7 or -0.5 to -0, 7) weak (0.3 to 0.5 or -0.3 to -0.5) and insignificant (0.0 to 0.3 or 0.0 to -0.3)^(^ 17 ^)^. For all statistical tests performed, a significance level of 5% (α=0.05) was used.

In order to comply with the determinations of Resolution nº 466/2012 of the National Health Council, this study was approved by the Research Ethics Committee from the University of São Paulo at Ribeirão Preto College of Nursing, under the number CAAE 63229816.5.0000.5393.

## Results

The percentage of respondents per municipality exceeded 60% for both the technicians’ category and the nursing coordinators. From the end of data collection, we obtained a population of 221 subjects, that is, a percentage of 75.11% (n=166) of research participants, with eleven coordinators and 155 nursing technicians. Among these, most (n=103, 62.04%) were female workers with a state employment bond (n=100, 60.24%).

Concerning the nursing coordinators, the average age was 38 years old, with standard deviation (SD) of 7.09. This population, almost entirely n=10 (90.91%), has specialization and meets an eight-hour scale daily. Their average training time was 10.45 years (SD=3.70) and working time in the unit was 4.73 years, with SD=2.80.

For nursing technicians, the average age was 39.87 years old (SD=8.17), that is, close to the average of nursing coordinators. For these professionals, the average time of training was 13.33 years (SD=6.20), while the length of service in the unit was 6.55 years (SD=3.63). When analyzing these professionals’ training, of the 155 professionals in this category, 87 (56.13%) have a complementary course (as a plaster technician or occupational safety nursing technician) and 95 (61.29%) are graduated in the most diverse higher education areas.

Considering the characterization of the professionals participating in the survey, Tables 1 and 2 respectively show the correlation between the dimensions of Coaching Leadership and job satisfaction in the self-perception of the coordinators and in the perception of nursing technicians. For each correlation among these dimensions, the value in the first line corresponds to Spearman (r) and, in the second one, to its *p*-value.

**Table 1:** Correlation between the dimensions of Coaching Leadership and job satisfaction, in the self-perception of nursing coordinators of SAMU* Goias, Brazil, 2017

		Dimensions of Coaching Leadership				
		Communication	Giving and receiving feedback	Empowering and influencing	Supporting the team in achieving results	Total scale score
Dimensions of job satisfaction	Salary	0.385^†^	0.367	0.205	0.121	0.392
		0.242^‡^	0.266	0.543	0.722	0.232
	Promotion	0.364	0.496	0.171	0.113	0.351
		0.270	0.120	0.613	0.739	0.288
	Supervision	0.395	0.670	0.687	0.636	0.761
		0.2282	0.023	0.019	0.035	0.006
	Benefits	0.597	0.603	0.322	0.180	0.450
		0.052	0.049	0.333	0.595	0.164
	Rewards	0.379	0.060	-0.161	0.596	0.409
		0.249	0.860	0.634	0.052	0.210
	Operational Protocols	0.137	0.209	0.054	0.325	0.399
		0.687	0.535	0.873	0.328	0.223
	Employees	0.510	0.550	0.563	0.721	0.761
		0.108	0.079	0.071	0.012	0.006
	Job Nature	0.417	0.202	0.367	0.171	0.314
		0.201	0.550	0.266	0.614	0.345
	Communication	0.350	0.445	0.622	0.427	0.659
		0.289	0.169	0.040	0.189	0.027
	Total scale score	0.682	0.695	0.490	0.634	0.835
		0.020	0.017	0.125	0.036	0.001

* Mobile Emergency Care Service;

† r - Spearman Test;

‡ Spearman *p*-value

**Table 2:** Correlation between the dimensions of Coaching Leadership and job satisfaction, in the perception of nursing technicians of SAMU* Goias, Brazil, 2017

		Dimensions of Coaching Leadership				
		Communication	Giving and receiving feedback	Empowering and influencing	Supporting the team in achieving results	Total scale score
Dimensions of job satisfaction	Salary	0.053^†^	0.187	0.116	0.168	0.151
		0.510^‡^	0.019	0.148	0.035	0.059
	Promotion	0.336	0.455	0.408	0.455	0.459
		<.000	<.000	<.000	<.000	<.000
	Supervision	0.595	0.540	0.637	0.511	0.616
		<.000	<.000	<.000	<.000	<.000
	Benefits	0.138	0.280	0.286	0.325	0.301
		0.084	0.000	0.000	<.000	0.000
	Rewards	0.332	0.441	0.434	0.477	0.472
		<.000	<.000	<.000	<.000	<.000
	Operational Protocols	0.247	0.211	0.205	0.157	0.214
		0.001	0.008	0.010	0.049	0.007
	Employees	0.215	0.315	0.223	0.210	0.268
		0.007	<.000	0.005	0.008	0.000
	Job nature	0.309	0.360	0.365	0.362	0.379
		<.000	<.000	<.000	<.000	<.000
	Communication	0.533	0.562	0.531	0.507	0.573
		<.000	<.000	<.000	<.000	<.000
	Total scale score	0.551	0.652	0.635	0.622	0.678
		<.000	<.000	<.000	<.000	<.000

* Mobile Emergency Care Service;

† r – Spearman Test;

‡ Spearman *p*-value

According to Table 1, it is found from the values of Spearman (r) and *p*-value related to the total scores of the two scales that the practice of Coaching Leadership performed by nursing coordinators positively correlated (r=0.835; *p*-value=0.00) with job satisfaction, according to the self-perception of these professionals. Thus, the JSS dimensions - “employees” and “communication” - showed a high correlation with the total score of the leadership scale.

“Supervision” obtained a moderate correlation with the “giving and receiving feedback” dimensions (r=0.670; *p*-value=0.023), “empowering and influencing” (r=0.687; *p*-value=0.019) and “supporting the team in achieving results” (r=0.636; *p*-value=0.035). In addition, there was a strong correlation between “supporting the team in achieving results” and “employees” (r=0.721; *p*-value=0.012). It was also found that three out of the four dimensions referring to Coaching Leadership practice - “communication” (r=0.682; *p*-value=0.020), “giving and receiving feedback” (r=0.695; *p*-value=0.017) and “supporting the team in achieving results” (r=0.634; *p*-value=0.036) - showed a moderate correlation with the total job satisfaction score. In the others, the correlations established between the dimensions of the two scales were weak, insignificant or without any correlation.

Table 2 shows a moderate correlation between the total scores of the two scales (r=0.678 and *p*-value<.0001), as well as among the four dimensions of the Coaching Leadership scale. Likewise, there was a moderate association (r=0.595 and *p*-value<.000) of this scale score with the “supervision” and “communication” dimensions of the job satisfaction scale. The other correlations established between the domains of the two instruments were weak, insignificant or nonexistent.

## Discussion

Based on the results of both tables, Coaching Leadership had a positive correlation with job satisfaction, answering the question of this research. It is possible to be seen from the analysis of the total scores of the scales, either from the self-perception of the coordinators (strong correlation) or the perception of nursing technicians (moderate correlation). These results are in line with studies on the subject, showing that contemporary leadership models establish a positive relationship between leadership and job satisfaction^(^ 5 ^-^ 6 ^)^.

In Table 1, based on Spearman coefficients and *p*-value, it was found that the self-perception of the coordinators in relation to the Coaching Leadership exercise showed a moderate correlation with some specific domains of job satisfaction, including “supervision”. Similarly, in the technicians’ perception (Table 2), there was a moderate correlation among the dimensions of this leadership model and the “supervision” component of the JSS instrument.

Thus, it is important to highlight a study conducted in a highly complex hospital in China which showed a statistically significant relationship between leadership and job satisfaction, especially in the field of “hospital supervision and policy”^(^ 18 ^)^.

In this same perspective, three researches published temporarily close, but developed in different continents, presented similar results. A study conducted in Portugal was based on the theoretical reference of situational leadership to determine its correlation with job satisfaction it was verified, through the data found, a greater relationship between leadership style (delegate) and “supervision”. The authors observed that supervisory behaviors are less present as a result of the greater relationship between leaders and other individuals in strongly mature groups^(^ 19 ^)^. A similar result was observed in a study conducted in Chile, which used the same theoretical reference^(^ 20 ^)^.

The third study, conducted in Ethiopia, adopted the transformational/transactional leadership reference to investigate its correlation with satisfaction. At the time, there was a strong correlation between transformational leadership and extrinsic aspects of satisfaction, in which “supervision” is emphasized^(^ 21 ^)^. Thus, it became evident that “supervision” is significant for nursing professionals.

Considering the emergency context, the same as this research, nurses need to understand how complex and primordial their supervisor role is in a Mobile Prehospital Care team. They should also understand that such supervision extends beyond the pursuit of qualification and recognition of its impact, not just to the design of supervisory action^(^ 22 ^)^.

In line with this aspect, when performing their supervisory role in their daily work process, nurses should not exercise it in an inarticulate manner. However, this professional should analyze the organizational context and its relations with health policies, monitor the interventions and their results, and also improve and qualify the work agents through more participative and democratic teaching-learning processes^(^ 23 ^)^.

Moreover, it is important to emphasize the correlation between the total score of Coaching Leadership and the “communication” domain, referring to job satisfaction, which, based on Tables 1 and 2, also had a statistically significant relationship. Thus, it should be stressed that in the action of communication, human interaction is considered as a fundamental element both in the process of leadership exercised by nurses and in the establishment of team bonds^(^ 24 ^)^.

Thus, health institutions need to promote leadership training in nursing to increase organizational structures and policies that support the effectiveness of communication and, therefore, provide a favorable workplace for job satisfaction^(^ 25 ^)^.

Moreover, analyzing the interpersonal relationship between leaders and the team, communication contemplates an important aspect for group satisfaction, for increasing teamwork and for performing activities^(^ 26 ^)^. The efficiency of communication by nurses allows the identification of individual and collective problems and, as a result, establish more efficient strategies for safe and qualified care, as well as for team satisfaction^(^ 27 ^)^.

Another highlighting point in Table 1 was the strong correlation between the “employees” and “supporting the team in achieving results” items. It shows that this research is in line with the Canadian study, which pointed out that leaders who show support for teamwork improve group relationships, promote emotions in the workplace and, consequently, increase the work effectiveness of nursing professionals and improve job satisfaction^(^ 28 ^)^.

Nurses who work in emergency units are permanently surrounded by challenges. These professionals need not only to motivate the workers of their team, but also to engage managers to make commitments and contribute to ensuring a favorable environment for nursing practices. In addition, it is through leadership that nurses will lead their team in a cohesive manner, encouraging mutual collaboration for qualified emergency care, which provides the best care for patients who need this service^(^ 29 ^)^.

Considering the existing data in the tables, it is evident the importance of leadership in prehospital care environments. Since the leading nurse is responsible for channeling the attention of those involved and directing them to common ideals, the actions of this professional aim to bring together, adjust group and individual interests in consensus with the organization’s objectives, affecting the quality of care^(^ 30 ^)^.

Thus, it is noted that the SAMU nursing coordinator fulfills an essential function on his team when exercising the leadership. This competency, based on the Coaching Leadership domains - communication, giving and receiving feedback, empowering and influencing and supporting the team - tends to provide a timely work environment for care to be delivered while concurrently contributing to a favorable environment regarding job satisfaction.

Based on the results of Tables 1 and 2, it is important to report that the success or failure in balancing the focus on production and/or guiding the people involved within the organization is due, among other factors, to the conversion of the exercise analysis of leadership by the leader, as well as by the team^(^ 31 ^)^.

It is observed that the level of relationship between leaders and their team members corresponds to a significant indicator to measure the leadership of an organization^(^ 32 ^)^. In this logic, in order to carry out studies of this nature that adequately cover the topic, it is necessary to use an instrument to evaluate this congruence, relevant to the theme of leadership in nursing, which considers the local institutional context and literature on the topic^(^ 31 ^)^.

As an example, it is worth mentioning the study which analyzed the ideal and real leadership style based on the nursing technician’s perception and the nurse’s view. The characteristics linked to the analyzed article converged to the same leadership style: a model that gives relevance not only to the aspects of production, but also to the relationship with people^(^ 2 ^)^.

In this perspective, the use of the QUAPEEL and QUEPTAEEL instruments are justified for leadership assessment, as well as the JSS for the job satisfaction analysis of the two categories of professionals, in order to allow the correlation among the variables. Finally, a pertinent point to consider is that leadership and satisfaction success is related not only to the leader’s behavior, but also to the relationship and engagement of the team members.

Regarding this aspect, it is appropriate to cite a research conducted in the state of Espirito Santo, Brazil, which from the use of two instruments for different professionals - nurses and nursing technicians - showed that 79% of nurses, in their self-perception, stated that the exercise of their leadership aimed at balancing the company’s objectives and the satisfaction of the workers in the sector. On the other hand, the results of the same assessment for nursing technicians responding to the study were inconclusive, as they divided their assessment between “neither disagree nor agree” and “agree” with the statement^(^ 31 ^)^.

Due to the relevance of nurses’ leadership, it is up to the nursing team leader (in the case of the nursing coordinator) to have a thoughtful view towards the teams regarding subjective issues in the work process: worker satisfaction, the positive relationship among group members, and others^(^ 24 ^)^.

In addition, it is worth noting that nursing has been conquering its space in SAMU through new knowledge. To do so, it is essential that the team ensures a good interpersonal relationship and that the nurse, when coordinating the nursing team, is able to explore opportunities for fruitful communication among team members. In addition, the team must have preparation, high level of knowledge, in order to promote for itself greater autonomy and security, consequently providing a feeling of satisfaction on them^(^ 33 ^)^.

The theoretical and practical knowledge associated with processes and routines in the context of emergencies is fundamental for the effective exercise of leadership. In this perspective, it is worth pointing out that these services require investments in skills such as communication, interpersonal relationships, among others, in order to achieve the exercise of nursing leadership^(^ 14 ^)^. Along with these investments, the leading nurse needs to influence and inspire the team, as well as strengthen a bond of harmony, trust and friendship, while motivating the group^(^ 29 ^)^.

Leadership is not only limited to a management function, but it is a competence that must be present in nursing professional practice. Thus, the development of excellence in nursing leadership is intrinsically associated with the approach of this competence since the first years of graduation, improving and intensifying throughout the career.

So, it is essential to rescue Coaching Leadership, a reference adopted in this research, as a reference model for the exercise of this competence by nurses. It corresponds to a set of attributes that can be systematically apprehended, used and improved, provided that the commitment and values declared and put into practice are not neglected^(^ 10 ^)^. In addition, Coaching Leadership is a work in constant progress^(^ 34 ^)^.

The results described in Table 2 allowed us to analyze which domains the coordinators should prioritize to enable job satisfaction of SAMU’s working nursing technicians. Similarly, a research conducted in the State of Amazonas with nursing technicians regarding the perception of the leadership of the nursing coordinator allowed the manager-leader nurse to know, from this perspective of nursing technicians, which management skills needed to be improved, contributing to as a guiding instrument for the self-assessment of the coordinator as a manager-leader nurse^(^ 35 ^)^.

In this context the nurse-coach leader, naturally, assumes the role of an educator by engaging in the training and enhancement of the team, developing skills and actions such as the sustainability of dialogue and the maintenance of clear communication, the expansion of the team’s self-knowledge, investigation of group evolution, stimulation of the team, exploration and development of curiosity, continuity of focus of team members, sharing of ideas and support, as well as authenticity and respect^(^ 10 ^)^. As a result, team members may be more committed to performing tasks and goals with the organization and, possibly, performing their activities and functions with greater satisfaction.

The analysis of the correlation between the two research variables made it possible to identify the main barriers to the satisfaction of technicians through their leadership. It also allowed identifying its potentialities and weaknesses, considering the four domains of Coaching Leadership and thus outline strategies to stimulate the satisfaction of the professionals led.

Currently, there is a lack of nurses with managerial and leadership profile in hospitals and health organizations. These professionals, once appointed as successful leaders, progressively engage with their work and their team members, think collectively and anticipate irrelevant conflicts, as they have the knowledge, attitudes and skills necessary for team management^(^ 36 ^)^.

In addition, it is important to highlight the planning act of the leading nurse, since when appropriate it favors to mitigate dissatisfaction and demotivation, contributes to cost reduction and increases productivity. However, in order to achieve such actions, the nurse must be involved, committed, communicated and effectively develop skills such as empathy to ensure more effective management^(^ 37 ^-^ 39 ^)^.

Based on the Coaching Leadership reference, it was evident from the data in both tables that such leadership model is linked to the existence of a favorable space marked by mutual trust, directly related to the interaction between leader (nurse) and his team members (nursing technicians) in pursuit of professional and personal development.

It is important to point out that the research participants could have a greater perception about this leadership model, because the nursing coordinators of SAMU units evaluated had no specific training or qualification. Thus, the evaluation of the professionals about the exercise of this competence was based on the dimensions of the Coaching Leadership process.

Besides the non-adherence of all technicians, another limiting point to this research is due to the fact that the study did not include nurses from SAMU Advanced Support Units (USA - abbreviation in Portuguese), since the instruments used were not built and validated for the analysis of nurses and their leaders. However, these aspects do not minimize the relevance of the study of this topic for nursing.

Therefore, it is important to highlight the implications arising from this study that aimed to add knowledge about management and nursing management, especially with regard to leadership in this area. It also aimed to analyze its relationship with the work environment, focusing on the job satisfaction variable, especially related to workers’ health quality. Finally, dimensioning the Coaching Leadership exercised by nursing coordinators in SAMU units makes it possible to direct actions and resources for the development of this competence in professionals working in the context of mobile prehospital care.

## Conclusion

From the data analysis, there was a statistically significant correlation between the Coaching Leadership and job satisfaction. In the self-perception of nursing coordinators, the *p*-value (0.001) and the Spearman coefficient (0.835) denote strong correlation, since in the nursing technicians’ perception the *p*-value (<.001) and the Spearman coefficient (0.678) implies a moderate correlation, demonstrating how the exercise of the Coaching Leadership by nursing coordinator influences the satisfaction of nursing technicians.

When more specifically assessed the correlation among the four existing domains of Coaching Leadership with total job satisfaction score, there was a correlation in almost all of them, except for the “empowering and influencing”, with “supervision”, both in the self-perception of the coordinators and in the analysis of nursing technicians.

Based on this, it was found that SAMU’s nursing coordinator needs to develop leadership, aiming to mitigate the barriers in the exercise and improve this competence, ensuring the satisfaction of professionals. For this reason, nowadays the presence of these professionals is essential for emergency care, since it makes possible to guarantee the group’s leadership, the technical improvement of the teams, the construction of care protocols and, finally, the team’s supervision.
